# Anatomical segmentectomy of the lung: tip of identifying the intersegmental plane

**DOI:** 10.1186/1749-8090-8-S1-O226

**Published:** 2013-09-11

**Authors:** M Kamiyoshihara, H Igai, T Ibe, N Kawatani, I Shiraishi, K Obayashi, S Nakazawa, Y Ohtaki, K Shimizu, I Takeyoshi

**Affiliations:** 1 Department of General Thoracic Surgery, Maebashi Red Cross Hospital, Maebashi, Japan; 2 Gunma University Graduate School of Medicine, Maebashi, Japan

## Background

A pulmonary segmentectomy requires identification of the segmental planes, making it technically more difficult than a lobectomy. Therefore, we present a selective segmental-inflation technique using a butterfly needle. This paper discusses anatomical segmenectomy with special reference to identifying the intersegmental plane.

## Methods

First, the lung is deflated and the pulmonary vessels to the involved segment are divided. The segmental bronchus is divided using a stapling device or ligation. Then, using a butterfly needle, oxygen (approximately 1 L/min.) is instilled into the targeted bronchus to inflate the involved segment, and the involved segment is severed and removed using electrocautery or a stapling device. The raw surface is covered with an absorbable sealing material to prevent air leaks.

## Results

Fifty-three (M:F = 33:33) patients underwent anatomical segmentectomy with the selective segmental-inflation technique using a butterfly needle. Their median age was 64.9 years. The diseases were malignant lung disease in 52 patients and benign lung disease in 14. The surgical procedure was segmentectomy only in 60 and segmentectomy combined with lobectomy in six. The median operating time was 170 min; the blood loss was 82 g; and the postoperative stay was 8 days. The duration of chest tube drainage was 3 days. No major complication occurred.

## Conclusions

In summary, anatomical segmentectomy was performed successfully with the selective segmental-inflation technique using a butterfly needle. Advantages: Surgeons can control every manipulation. No special device is needed; a butterfly needle is sufficient. It is useful regardless of the situation of proximal bronchus. Ultra-selective air instillation into the subsegmental bronchus depends on the needle direction. Disadvantages: The proximal site of the targeted bronchus must be identified. Care is needed to avoid systemic air embolism.

